# Celecoxib Ameliorates Portal Hypertension of the Cirrhotic Rats through the Dual Inhibitory Effects on the Intrahepatic Fibrosis and Angiogenesis

**DOI:** 10.1371/journal.pone.0069309

**Published:** 2013-07-26

**Authors:** Jin-Hang Gao, Shi-Lei Wen, Wen-Juan Yang, Yao-Yao Lu, Huan Tong, Zhi-Yin Huang, Zhang-Xu Liu, Cheng-Wei Tang

**Affiliations:** 1 Division of Peptides Related with Human Diseases, State Key Laboratory of Biotherapy, West China Hospital, Sichuan University, Chengdu, China; 2 Department of Gastroenterology, West China Hospital, Sichuan University, Chengdu, China; 3 Regenerative Medicine Research Center, West China Hospital, Sichuan University, Chengdu, China; 4 Research Center for Liver Diseases, Keck School of Medicine, University of Southern California, Los Angeles, California, United States of America; Faculty of Pharmacy, Ain Shams University, Egypt

## Abstract

**Background:**

Increased intra-hepatic resistance to portal blood flow is the primary factor leading to portal hypertension in cirrhosis. Up-regulated expression of cyclooxygenase-2 (COX-2) in the cirrhotic liver might be a potential target to ameliorate portal hypertension.

**Objective:**

To verify the effect of celecoxib, a selective inhibitor of COX-2, on portal hypertension and the mechanisms behind it.

**Methods:**

Cirrhotic liver model of rat was established by peritoneal injection of thiacetamide (TAA). 36 rats were randomly assigned to control, TAA and TAA+celecoxib groups. Portal pressures were measured by introduction of catheters into portal vein. Hepatic fibrosis was assessed by the visible hepatic fibrotic areas and mRNAs for collagen III and α-SMA. The neovasculature was determined by hepatic vascular areas, vascular casts and CD31 expression. Expressions of COX-2, vascular endothelial growth factor (VEGF), VEGF receptor-2 (VEGFR-2) and related signal molecules were quantitated.

**Results:**

Compared with TAA group, the portal pressure in TAA+celecoxib group was significantly decreased by 17.8%, *p*<0.01. Celecoxib treatment greatly reduced the tortuous hepatic portal venules. The data of fibrotic areas, CD31expression, mRNA levels of α-SMA and collagen III in TAA+celecoxib group were much lower than those in TAA group, *p*<0.01. Furthermore, the up-regulation of hepatic mRNA and protein levels of VEGF, VEGFR-2 and COX-2 induced by TAA was significantly inhibited after celecoxib treatment. The expressions of prostaglandin E2 (PGE2), phosphorylated extracellular signal-regulated kinase (p-ERK), hypoxia-inducible factor-1α (HIF-1α), and c-fos were also down-regulated after celecoxib treatment.

**Conclusions:**

Long term administration of celecoxib can efficiently ameliorate portal hypertension in TAA rat model by its dual inhibitory effects on the intrahepatic fibrosis and angiogenesis. The anti-angiogenesis effect afforded by celecoxib may attribute to its modulation on VEGF/VEGFR-2 through the down-regulation of integrated signal pathways involving PGE2- HIF-1α- VEGF and p-ERK- c-fos- VEGFR-2.

## Introduction

Portal hypertension, a major complication of advanced liver cirrhosis [1], is the result of increased vascular resistance in the portal circulation, increased portal venous blood flow, or both. Its life-threatening consequences carry a poor prognosis and represent the first cause of death and need for liver transplantation in patients with cirrhosis [2]. Unfortunately, the efficacy of pharmacological therapy aimed at correcting the increased splanchnic blood flow is limited to control portal hypertension.

Fibrosis, regenerative nodule formation, and intrahepatic vasoconstriction are classical mechanisms that account for increased intrahepatic vascular resistance in cirrhosis. Recent data suggest that intrahepatic angiogenesis could also be involved in fibrosis and portal hypertension [2], [3]. Liver cirrhosis is closely associated with increased angiogenesis, and the neovasculature is mainly located in the fibrotic areas of the liver [4]–[6]. Angiogenesis refers to the development of new blood vessels from the existing vasculature [7]. In support of this, vascular endothelial growth factor (VEGF), placental growth factor and angiopoietin I have been found to be over expressed in cirrhotic liver [5], [8]–[10]. Anti-angiogenesis strategy has recently emerged as an actively investigated treatment for liver cirrhosis [11]. Several angiogenesis inhibitors, such as bevacizumab, sorafenib, sunitinib, pazopanib and everolimus, have been approved or tested in the treatment of hepatocellular carcinoma [12] but not for the application in the patients with liver cirrhosis because of their toxicities and side effects [13], [14].

Cyclooxygenase-2 (COX-2) is a rate-limiting enzyme involved in the conversion of arachidonic acid to prostaglandins (PGs) and thromboxanes [15]. COX-2 expression is up-regulated in the cirrhotic liver [16]. COX-2 inhibitors can reduce angiogenesis in hepatocellular carcinoma and other tumors [17]–[20]. However, the effect of selective COX-2 inhibitors on the angiogenesis in cirrhotic liver remains unclear. Celecoxib, a selective COX-2 inhibitor, is safe and has been widely applied in the treatment of rheumatic diseases. In this study, we investigated the antiangiogenic and antifibrotic effects of celecoxib on the development of liver cirrhosis in rats treated with thiacetamide (TAA).

## Materials and Methods

### Animal Care

All the rats used in this study were kept under a 12 h light-dark cycles at a constant temperature and humidity with free access to chow and water. The animal procedures were approved by the Animal Use and Care Committee of Sichuan University and were conducted according to the regulations set by Sichuan University.

### Animals and Experimental Designs

Peritoneal injection (i.p.) of TAA (Sigma Chemical Co., St. Louis, MO, USA) was employed to induce liver cirrhosis (200 mg/kg every 3 days for 16 weeks). 36 male Sprague-Dawley rats (Experimental Animal Center of Sichuan University, Chengdu, China), weighing 180 to 230 g, were randomized into control, TAA and TAA+celecoxib groups with 12 animals in each group. TAA+celecoxib group received TAA plus celecoxib (20 mg/kg/day, Pfizer, New York, NY, USA) by gastric gavage from the initiation of TAA administration. TAA group received TAA plus placebo and control group received injections of normal saline (1mL i.p., every 3 days). The rats in each group were sacrificed under anesthesia at the end of experiments. Portions of liver tissues were fixed in 4% neutral buffered paraformaldehyde for histopathologic and immunohistochemical examinations, or immediately frozen in liquid nitrogen and stored at −80°C for further RNA and protein analysis. Serum was collected and stored at −80°C until analysis.

### Histopathological Study of Liver Cirrhosis and Angiogenesis

Liver tissues fixed in 4% paraformaldehyde were embedded in paraffin, sectioned (thickness of 6 µm), and then stained with hematoxylin and eosin (HE) and Masson trichrome (MT). The morphological changes were examined under a microscope (CX41, Olympus, Tokyo, Japan) equipped with a digital camera (DP72, Olympus, Tokyo, Japan) and analyzed by Image-Pro Plus 4.0 software (Media Cybernetics, Silver Spring, MD, USA). The vascular areas were measured on HE staining sections at ×100 magnifications. The percentages of fibrotic areas were calculated for each MT-stained section at ×100 magnifications. To avoid the sampling bias in the semi-quantitative analysis, a total of 12 pieces of liver tissues from each animal were obtained (3 pieces from each of the left lateral, right medial and right lateral lobes; 1 piece from each of the other 3 small lobes).

### Vascular Corrosion Casting and Scanning Electron Microscope (SEM) of Liver Vasculature

Liver vascular corrosion casts, which explore the three dimensional morphology of the hepatic microcirculation, were performed in three rats from each group. The rats were deeply anesthetized with chloral hydrate. The livers were first perfused with heparin saline solution (contains 500 U/mL of heparin) through a cannula placed in the portal vein. After that, the vasculature of the rat livers were perfused in situ via the portal vein with a freshly prepared solution of methyl methacrylate (Sigma Chemical Co., St. Louis, MO, USA) containing 0.2 g of benzoyl peroxide (Sigma Chemical Co., St. Louis, MO, USA) per 100 mL of resin. After perfusion, the livers were excised and placed in a water bath for 72 hours to complete the polymerization of the perfused resin, and then the livers were immersed in 15% potassium hydroxide to digest the tissue off the cast, which was usually achieved in 4 to 5 weeks. The vascular casts were washed in running water and air-dried. Finally, the liver vascular corrosion casts were coated with gold and examined by SEM (JSM-7500F, JEOL, Japan).

### Immunohistochemical Studies for Liver Sections

Liver tissues were processed for paraffin embedding and 3 µm of sections. The sections were deparaffinized in xylene and serial ethanol dilutions. Antigen retrieval was performed by heating the sections in 10 mM sodium citrate buffer. Sections were blocked and incubated with primer antibody overnight at 4°C and developed with biotinylated secondary antibodies and then incubated with streptavidin-biotin-complex. The sections were then stained with a solution of 3, 3-diaminobenzidine tetrahydrochloride and counterstained with haematoxylin. The positive areas of VEGF (1:100, Abcam, Cambridge, UK), COX-2 (1:150, Santa Cruz Biotechnology, Santa Cruz, CA, USA), phosphorylated extracellular signal-regulated kinase (p-ERK, 1:100, Signalway Antibody, Pearland, TX, USA) and VEGF receptor 2 (VEGFR-2, 1:100, Bioss, Beijing, China) were quantified by using Image-Pro Plus 4.0 software to score integrated optical density (IOD) at ×400 magnifications. For the quantification of sections stained for CD31 (1:200, Bioss, Beijing, China), five random fields at ×400 magnifications were captured and the percentages of CD31-positive areas were calculated for each liver section.

### Western Blot Analysis for Hepatic Protein Expression

Proteins were extracted from the livers using a protein extraction kit (Nanjing Kaiji, Nanjing, China) following guidelines from the manufacturer. 100 µg of proteins were electrophoresed on 10% SDS-PAGE. The gels were then blotted onto a nitrocellulose membrane (Millipore, Bedford, MA, USA). Non-specific binding sites in the membranes were blocked with 5% non-fat dry milk in incubation buffer before addition of antibodies against rat VEGF (1:500), VEGFR-2 (1:400), CD31 (1:200), COX-2 (1:400) and β-actin (1:200, Bioss, Beijing, China). The blots were washed and incubated for one hour at room temperature with appropriate HRP-conjugated secondary antibodies. Protein bands were visualized by using an ECL detection kit (Amersham Pharmacia, Uppsala, Sweden). The autoradiographs were scanned after exposing the membranes to Kodak XAR film (Eastman Kodak, Rochester, NY, USA). Protein expression was determined by using Quantity One software 4.5.0 (Bio-Rad Laboratories, Hercules, CA, USA). The housekeeping protein β-actin was used as an internal control.

### Quantitative Real-time PCR (qRT-PCR) for Hepatic mRNA Expression

Total RNA was extracted from rat liver tissues by using TRIzol reagent (Invitrogen, Carlsbad, CA, USA). 5 µg of RNA was reverse-transcribed using a first-strand cDNA kit with random hexamers (Fermentas, Burlington, Canada) and qRT-PCR performed by using the SsoFast EvaGreen PCR master mix (Bio-Rad, Hercules, CA, USA). Intron-spanning primers are listed in Table 1. All reactions were run in duplicate by using a CFX96 real-time PCR detection system (Bio-Rad, Hercules, CA, USA). The mRNA expression was normalized to glyceraldehyde-3-phosphate dehydrogenase (GAPDH) by using the 2^−ΔΔCt^ method and shown as fold changes.

**Table 1:** List of primers for qRT-PCR.

Gene	GenBank Accession No.	Forward sequence (5′–3′)	Reverse sequence (5′–3′)	Expected Product Size
***GAPDH***	NM_017008	TCGGTGTGAACGGATTTG	CTCAGCCTTGACTGTGCC	173 bp
***CD31***	NM_031591	CTTCACCATCCAGAAGGAAGAGAC	CACTGGTATTCCATGTCTCTGGTG	360 bp
***COX-2***	NM_017232	GCTCATACTGATAGGAGAGACGA	TGGAACTGCTGGTTGAAAAG	117 bp
***VEGF***	NM_031836	CGAACGTACTTGCAGATGTGAC	GACGGTGACGATGGTGGT	139 bp
***VEGFR-2***	NM_013062	CATAATAGAAGGCGTCCAGG	GCTCATCCAAGGGCAGTT	191 bp
***HIF-1α***	NM_024359	TTCGGCAGCGATGACAC	CAGAGGCAGGTAATGGAGAC	130 bp
***c-fos***	NM_022197	AACACACAGGACTTTTGCG	CTCTGGTCTGCGATGGG	142 bp
***Collagen III***	NM_032085	GATGGCTGCACTAAACACACT	CACTTTCACTGGTTGACGAGA	241 bp
***α-SMA***	NM_031004	CCGAGATCTCACCGACTACC	TCCAGAGCGACATAGCACAG	120 bp

HIF-1α, Hypoxia-inducible factor-1α;

α-SMA, alpha-smooth muscle actin.

### Enzyme-Linked Immunosorbent Assay (ELISA) for Serum Prostaglandin E2 (PGE2)

The concentration of serum PGE2 was quantified with an ELISA kit according to the manufacturer’s instructions (USCN Life Sciences Inc, Wuhan, China). Plates were read using the Thermo microplate reader (Thermo Fisher scientific, San Jose, CA, USA) under the wavelengths of 450 nm.

### Hemodynamic Studies of Portal Pressure and Mean Artery Pressure

At the end of respective treatments, hemodynamic measurements were performed under chloral hydrate anaesthesia. PE-50 catheters were introduced into the carotid artery and portal vein separately to measure mean arterial pressure, heart rate and portal pressure. The measurements were recorded on a multichannel computer-based recorder BL420 (Taimeng Technology Co., Chengdu, China).

### Serum Biochemistry for Liver and Renal Function Parameters

The serum was collected and analyzed for liver and renal function parameters by an Olympus AU2700 analyser (Olympus, Tokyo, Japan), according to standard tests based on the recommendations of the International Federation of Clinical Chemistry.

### Statistical Analysis

All data were expressed as mean ± SD and analyzed by SPSS 13.0 software (SPSS, Chicago, IL, USA). One-way ANOVA test was applied in the comparisons among three groups. Then, a stepwise multiple comparisons procedure, Student-Newman-Keuls test was used to identify sample means that were significantly different from each other. A value of *p*<0.05 was considered significant.

## Results

### Successful Cirrhotic Liver Model Induced by TAA Treatment

**Figure 1:**
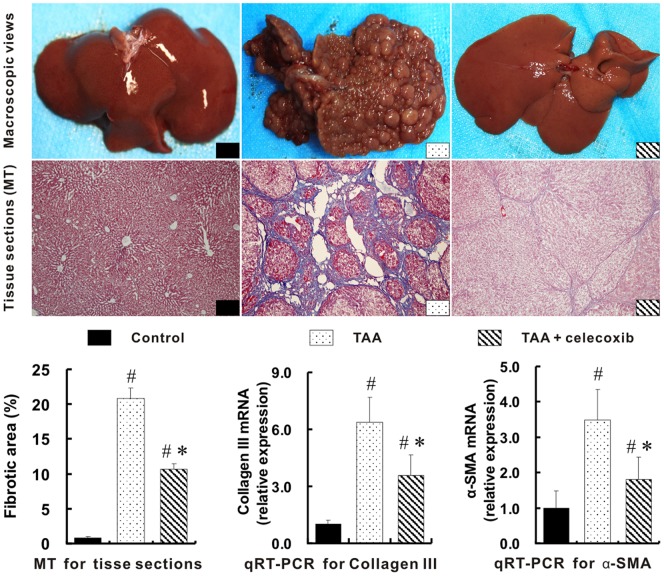
Reduction of hepatic fibrosis with celecoxib treatment. Compared with the livers in control group, a typical cirrhotic appearance with extensive nodular formation (middle of upper panel) was presented in the livers of TAA group. These hepatic nodules in TAA+celecoxib group were almost not observed by macroscopic view and were greatly reduced in the tissue sections (x100 magnifications) stained with MT. The percentages of fibrotic areas and expression of collagen III and α-SMA are plotted on the bottom. # *p*<0.01 *vs*. control group; * *p*<0.01 *vs*. TAA group.

Compared with the livers in control group, a typical cirrhotic appearance with extensive nodular formation was presented in the livers of rats treated with TAA for 16 weeks. Little or mild intrahepatic inflammatory infiltration was detected in these cirrhotic rats. MT staining sections (Figure 1) further revealed the fibrotic nodules and the distortion of hepatic structure in the rat livers of TAA group. The fibrotic area of liver tissues in TAA group were dramatically increased by 25 folds when compared with that of control group, *p*<0.01. Moreover, hepatic mRNAs for collagen III and alpha smooth muscle actin (α-SMA) in TAA model were 5.3 and 2.5 folds higher than those in control group, *p*<0.01.

One rat died in each of the TAA and TAA+celecoxib group while no death was observed in control group. No significant differences in the serum levels of ALT, AST, total protein, albumin, bilirubin, urea and creatinine were observed among the three groups, *p*>0.05 (Table 2).

**Table 2:** Effects of celecoxib on the liver and renal functions.

	Control	TAA	TAA+Celecoxib
ALT (IU/L)	67.9±15.6	78.2±8.1	82.0±17.6
AST (IU/L)	196.4±25.4	215.2±34.0	220.7±28.6
Total protein (g/L)	53.9±4.8	53.3±3.1	53.5±3.1
Albumin (g/L)	34.5±3.6	31.5±2.7	34.5±2.7
Bilirubin (µmol/L)	1.5±0.6	1.4±0.6	1.7±1.0
Urea (mmol/L)	8.07±1.68	8.92±2.61	7.80±1.59
Creatinine (µmol/L)	55.5±16.5	45.6±11.7	52.0±12.7

ALT, alanine aminotransferase; AST, aspartate aminotransferase.

### Reduction of Hepatic Fibrosis with Celecoxib Treatment

The hepatic nodules induced by TAA were almost not observed in TAA+celecoxib group (Figure 1, upper panel). The hepatic histopathological lesions were also substantially reduced in TAA+celecoxib group. In support of this, the fibrotic areas of liver tissues in TAA+celecoxib group were significantly decreased by 49% when compared with TAA group (10.6±0.9% *vs*. 20.8±1.5%. *p*<0.01). The up-regulated mRNAs for collagen III and α-SMA with TAA treatment were also greatly decreased with the addition of celecoxib (Figure 1).

### Inhibition of Hepatic Neoangiogenesis with Celecoxib Treatment

**Figure 2:**
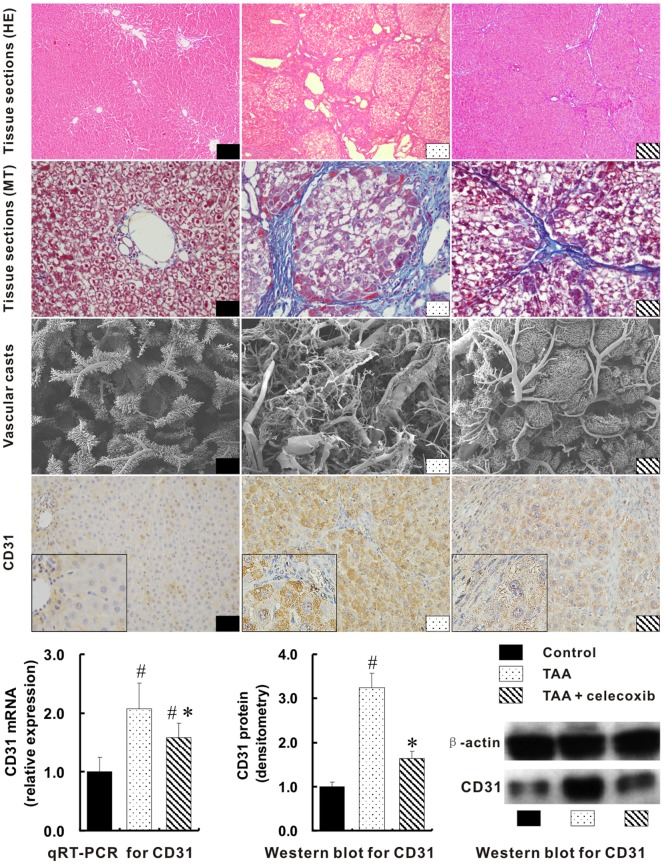
Inhibition of hepatic neoangiogenesis with celecoxib treatment. Hepatic vascular areas were shown with tissue sections stained by HE (×100 magnifications) and MT (×400 magnifications). The three-dimensional portal veins were visualized by vascular casts (SEM, ×35 magnifications). The expressions of hepatic CD31 were shown by immunohistochemical staining (4th panel, ×400 magnifications; inserts ×1000 magnifications). The mRNA and protein levels of hepatic CD31 were quantified by qRT-PCR and Western blot respectively (the bottom panel). # *p*<0.01 *vs*. control group; * *p*<0.01 *vs*. TAA group.

Tissue sections stained with HE and MT revealed that hepatic vascular areas in TAA group were greatly enlarged when compared with those in control group (0.0325±0.0086 mm^2^ *vs.* 0.1502±0.0143 mm^2^, *p*<0.01) (Figure 2). Such findings were further confirmed by the three-dimensional changes of portal veins. Vascular casts showed much more irregular, tortuous portal veins in the livers of TAA group. Correspondingly, the up-expression of hepatic CD31 in TAA group was visualized by immunohistochemistry. Furthermore, mRNA and protein levels of CD31 in TAA group were over one fold higher than those in control group, *p*<0.01 (Figure 2).

Interestingly, beside the vascular areas in TAA+celecoxib group were greatly reduced (0.1502±0.0143 mm^2^ *vs.* 0.0485±0.0097 mm^2^, *p*<0.01), less irregular and tortuous hepatic portal veins were showed in TAA+celecoxib group by vascular casts when compared with TAA group (Figure 2). Consistently, the down-expression of hepatic CD31 in TAA+celecoxib group was shown by immunohistochemistry. Quantitatively, the hepatic CD31 mRNA and proteins levels in TAA+celecoxib group were remarkably lower than those in TAA group, *p*<0.01 (Figure 2).

### Suppression of Angiogenesis through the Integrated Signal Pathways with Celecoxib Treatment

**Figure 3:**
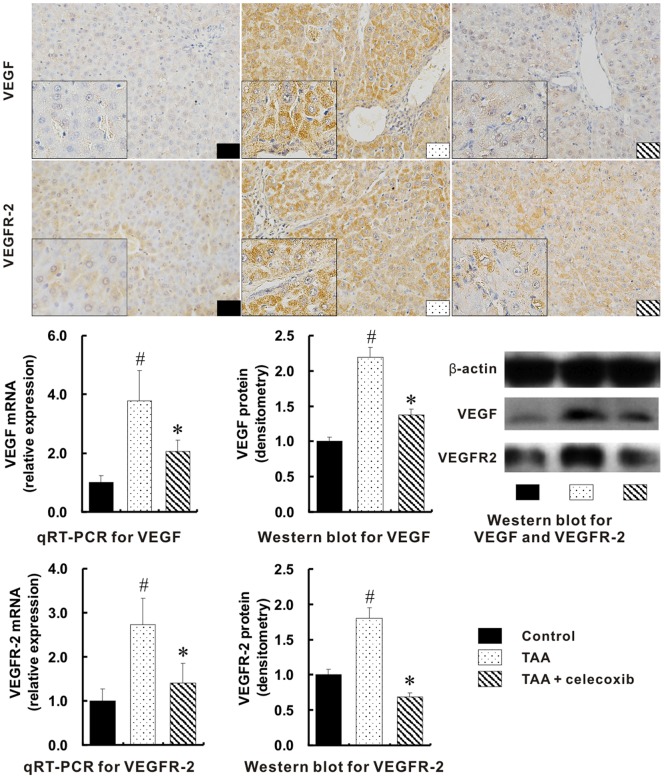
Suppression of angiogenetic factors by celecoxib treatment. Much more positive expressions for VEGF and VEGFR-2 were visualized in TAA group (1st - 2nd panels, Immunohistochemical stain, ×400 magnifications; inserts, ×1000 magnifications). The mRNA and protein levels of hepatic VEGF and VEGFR-2 in TAA group measured with qRT-PCR and Western blot respectively were the highest among three groups. # *p*<0.01 *vs*. control group; * *p*<0.01 *vs*. TAA group.

Immunohistochemical staining showed that the up-expressions of VEGF and VEGFR-2 in the liver of TAA group were markedly decreased with the addition of celecoxib (Figure 3). Consistently, the mRNA and protein quantifications of hepatic VEGF and VEGFR-2 in TAA group were increased over one-fold when compared with those in control group, but were significantly reduced with the celecoxib treatment (Figure 3).

**Figure 4:**
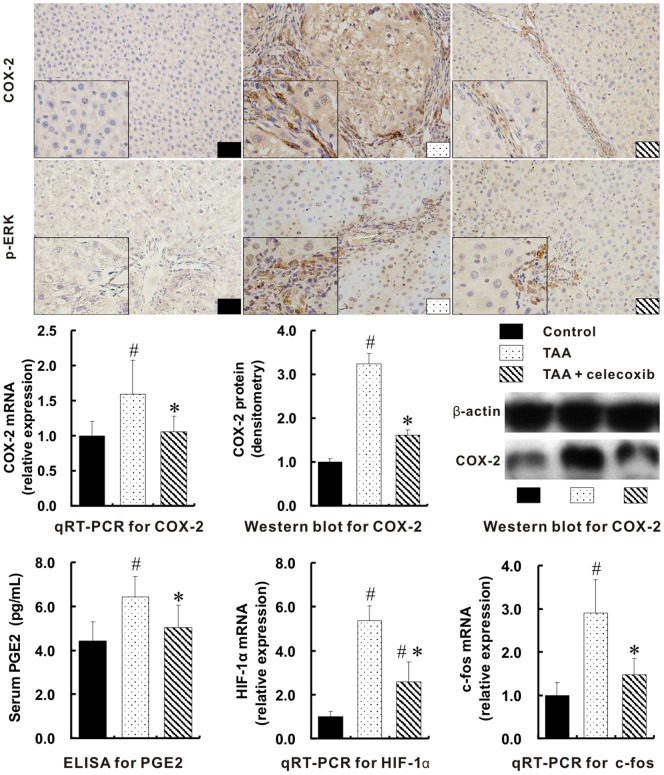
Suppression of the integrated signal pathways with celecoxib treatment. Much more positive expressions for COX-2 and p-ERK were visualized in TAA group (Immunohistochemical stain, ×400 magnifications; inserts, ×1000 magnifications). The mRNA and protein levels of hepatic COX-2 in TAA group measured with qRT-PCR and Western blot separately were the highest among three groups. The serum levels of PGE2 were measured by ELISA. The mRNAs for HIF-1α and c-fos were determined by qRT-PCR. # *p*<0.01 *vs*. control group; * *p*<0.01 *vs*. TAA group.

The hepatic mRNA for COX-2 in TAA group was the highest among three groups (*p*<0.01). The up-regulation of COX-2 protein in TAA group was either shown by immunohistochemical staining or measured by Western blot when compared with control group. With the addition of celecoxib, the hepatic COX-2 protein was dramatically down-regulated (Figure 4, *p*<0.01).

As the product of COX-2 protein, the serum concentration of PGE2 in TAA group was also the highest among three groups (*p*<0.01). Consequently, the mRNA expression of hepatic hypoxia-inducible factor-1α (HIF-1α), one of the downstream molecules of PGE2 and stimulator of VEGF, was significantly increased in TAA group when compared with that in control group, but was greatly decreased by the celecoxib treatment, *p*<0.01 (Figure 4,) In addition, hepatic p-ERK/c-fos pathway, a link between ERK and VEGF, was greatly activated in TAA model (*p*<0.01), but was suppressed by the addition of celecoxib (*p*<0.01).

### Decrease of Portal Hypertension by Celecoxib Treatment

**Figure 5:**
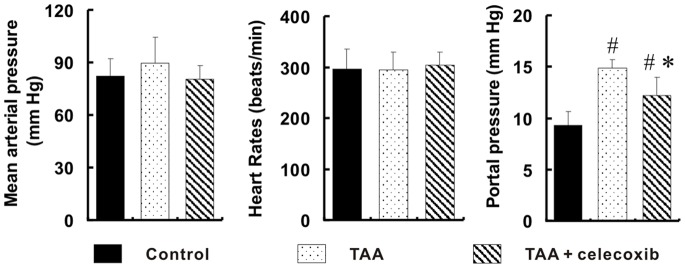
Decrease of portal hypertension by celecoxib treatment. There were no significant differences of the mean arterial pressure and heart rate among three groups, *p*>0.05. The portal pressure measured by introduced catheters in TAA group was the highest among three groups. # *p*<0.01 *vs*. control group; * *p*<0.01 *vs*. TAA group.

There were no significant differences of the mean arterial pressure and heart rate among three groups, *p*>0.05 (Figure 5). However the portal pressure in TAA group was significantly increased by 60.7% (14.88±0.84 mmHg *vs*. 9.26±1.39 mmHg, *p*<0.01) when compared with that in control group. It is valuable that the portal pressure in TAA+celecoxib group was significantly decreased by 17.8% (14.88±0.84 mmHg *vs*. 12.23±1.09 mmHg, *p*<0.01) (Figure 5).

## Discussion

Over the past two decades, significant advances in understanding of the molecular and cellular mechanisms underlying the development of liver cirrhosis have been achieved. It is generally accepted that activation of hepatic stellate cells (HSCs) and deposition of extracellular matrix are the driving forces leading to liver fibrosis and cirrhosis [21]. However, to date, many specific anti-fibrotic treatments have been tried, including inhibition of collagen synthesis, interruption of matrix deposition, stimulation of matrix degradation, modulation of HSCs activation, or induction of HSCs death, but none has succeeded yet [22], [23]. The cirrhotic liver model induced by TAA treatment in this study showed up-regulation of COX-2. By sufficiently inhibiting it for 16 weeks, celecoxib at the routinely recommended dosage effectively reduced visible hepatic fibrotic area and down-regulated hepatic mRNAs for collagen III and α-SMA. The mechanism underlying the anti-fibrogensis effect of celecoxib may be considered as induction of HSCs apoptosis through inhibition of Akt activation [24].

Liver cirrhosis is inevitable ultimate outcome triggered by consistent chronic liver injury and inflammation. Chronic inflammation is usually accompanied by angiogenesis [8]. Angiogenesis is a dynamic process leading to the formation of new vessels from pre-existing blood vessels, by sprouting or intussusception, then lumen formation and eventually stabilization of nascent vessels [25]. Recently, there is growing evidences supporting a causative role for angiogenesis in the development of fibrosis and portal hypertension in animal models [3], [10]. The results from this study also suggested that angiogenesis directly correlated with the degree of hepatic fibrosis. Indeed, intrahepatic angiogenesis and fibrosis developed in parallel in the TAA rat model. Several studies have administered anti-angiogenic drugs in animal models of cirrhosis with the aim of decreasing liver fibrosis and portal hypertension. The effect and benefit of anti-angiogenic interventions on the development of liver cirrhosis remain still controversial in the experimental models [26], [27]. Similar to anti-angiogenesis in hepatocellular carcinoma [17]–[20], this study showed an obvious inhibitory effect of celecoxib on the angiogenesis in the cirrhotic livers induced by TAA in terms of the reduced hepatic vascular areas, less tortuous portal veins displayed by vascular casts, and down-expression of hepatic CD31.

Of the numerous angiogenic factors discovered thus far, VEGF has been identified as a key mediator of neoangiogenesis. Elevated expression of VEGF in the cirrhotic livers of this study further indicated that liver cirrhosis is accompanied by angiogenesis and it is rational to suppress intrahepatic VEGF expression. Similar to sorafenib, which down-regulated the activity and expression of VEGF and VEGFR2 in mesenteries [26], celecoxib significantly reduced intrahepatic VEGF and VEGFR-2 by 37% and 62%, respectively. Among the integrated signal pathways linking COX-2 and VEGF, both PGE2 produced by COX-2 and its downstream molecular, HIF-1α, was greatly inhibited in the cirrhotic livers by celecoxib. HIF-1α is generally considered as one of the potential transcription factor-binding sites for VEGF. p-ERK is considered as essential inducer for VEGF receptor expression [28]. Up-regulation of p-ERK- c-fos pathway by COX-2 activation was observed in TAA group. Co-treatment with celecoxib led to down-regulation of p-ERK/c-fos pathway which could at least partly explain its inhibitory effect on VEGFR-2 expression.

Portal hypertension, a major complication of liver cirrhosis, can cause lethal complications, such as gastroesophageal variceal hemorrhage, ascites, hepatorenal syndrome and hepatic encephalopathy [1]. Historically treatment of portal hypertension has been mainly committing to cope with those complications. Currently, more attention has been paid to the intrahepatic anti-angiogenesis because portal hypertension is initiated in large part through increases of intrahepatic vascular resistance. However, few agents with a potential to reduce intrahepatic neovasculature have been used in the treatment of portal hypertension. On the basis of excluding the discrepancy of heart output among three groups, the present study verified the protective effect of celecoxib on the portal pressure in the TAA-treated rats. Furthermore, the morphological evidence of portal venules in three-dimension displayed by vascular casts among three groups provided an important tool to evaluate the relationship between portal hypertension and liver angiogenesis. Compared with the intuitively normal architecture of portal venules TAA treatment resulted in a tortuous vascular network of varying diameter and flow pattern, which were organized into micronodules and macronodules in the cirrhotic liver. Such profound changes clearly increased the inflow resistance and enhanced portal pressure about 60% in the rats treated with TAA. With the addition of celecoxib, the portal hypertension was significantly decreased most likely due to the amelioration of intrahepatic angiogenesis and fibrosis.

In contrast to the results in this study, some studies arguing against the effects of celecoxib on anti-fibrosis have been reported [29], [30]. The experiment term of cirrhotic model used in Yu’s group was quite shorter than that in our study, 2 weeks *vs.* 16 weeks of TAA treatment. Thus the data provided from Yu’s group, such as ALT, tumour necrosis factor-α, interleukin (IL)-1β and IL-6, mainly reflect a sub-acute inflammation other than typical chronic one. Differently, they did not examine the intrahepatic angiogenesis as one of the outcomes. In their study, the hepatic expression of COX-2 was not observed and its downstream eicosanoids was not inhibited by celecoxib treatment. The discrepancies between these two studies may suggest the better protective efficacy of celecoxib in the development of liver cirrhosis could be achieved in the setting of the chronic term with an extended treatment of TAA. Another study reported a detrimental role of celecoxib by showing that celecoxib exacerbates hepatic fibrosis and induces hepatocellular necrosis in rats treated with porcine serum [31], which may suggest that the effect of celecoxib in the prevention of liver cirrhosis might be dependent on the etiological differences of animal models.

Celecoxib has been widely used in the clinical treatment of osteoarthritis and rheumatoid arthritis [32]. A review of controlled clinical trials involving 6376 patients suggests that celecoxib has a very low risk for hepatotoxicity, even after exposures of as long as 2 years at therapeutic doses [33]. In this study, the biochemical parameters including liver and renal tests were comparable in three groups (Table 2), indicating that the long term use of celecoxib did not exacerbate hepatic and renal injuries in this model. Our results suggest that celecoxib is safe and efficacious in the prevention of liver cirrhosis in this animal model.

In conclusion, long term administration of celecoxib at the routinely recommended dosage can efficiently ameliorate portal hypertension in TAA rat model by its dual inhibitory effects on the intrahepatic fibrosis and angiogenesis. The anti-angiogenesis effect afforded by celecoxib may attribute to its modulation on VEGF/VEGFR-2 through the down-regulation of integrated signal pathways involving PGE2- HIF-1α- VEGF and p-ERK- c-fos- VEGFR-2. Taken together, our results suggest that celecoxib might be considered as a potential agent in the preventive strategy of liver cirrhosis for the patients with chronic hepatic diseases.
